# Roles of Protein Post-Translational Modifications During Adipocyte Senescence

**DOI:** 10.7150/ijbs.86404

**Published:** 2023-10-16

**Authors:** Min-Seon Hwang, Jingyeong Park, Yunha Ham, In Hye Lee, Kyung-Hee Chun

**Affiliations:** 1 Department of Biochemistry & Molecular Biology, Graduate School of Medical Science, Brain Korea 21 Project, Institute of Genetic Science, Yonsei University College of Medicine, 50-1 Yonsei-ro, Seodaemun-gu, Seoul, 03722, Republic of Korea.; 2 Department of Life Science, College of Natural Science, Ewha Womans University, 52 Ewhayeodae-Gil, Seodaemun-gu, Seoul, 03760, Republic of Korea.

**Keywords:** Adipocyte, Senescence, Post-translational modification, Metabolic disease, Metabolic homeostasis

## Abstract

Adipocytes are adipose tissues that supply energy to the body through lipids. The two main types of adipocytes comprise white adipocytes (WAT) that store energy, and brown adipocytes (BAT), which generate heat by burning stored fat (thermogenesis). Emerging evidence indicates that dysregulated adipocyte senescence may disrupt metabolic homeostasis, leading to various diseases and aging. Adipocytes undergo senescence via irreversible cell-cycle arrest in response to DNA damage, oxidative stress, telomere dysfunction, or adipocyte over-expansion upon chronic lipid accumulation. The amount of detectable BAT decreases with age. Activation of cell cycle regulators and dysregulation of adipogenesis-regulating factors may constitute a molecular mechanism that accelerates adipocyte senescence. To better understand the regulation of adipocyte senescence, the effects of post-translational modifications (PTMs), is essential for clarifying the activity and stability of these proteins. PTMs are covalent enzymatic protein modifications introduced following protein biosynthesis, such as phosphorylation, acetylation, ubiquitination, or glycosylation. Determining the contribution of PTMs to adipocyte senescence may identify new therapeutic targets for the regulation of adipocyte senescence. In this review, we discuss a conceptual case in which PTMs regulate adipocyte senescence and explain the mechanisms underlying protein regulation, which may lead to the development of effective strategies to combat metabolic diseases.

## Introduction

**Figure 1:**
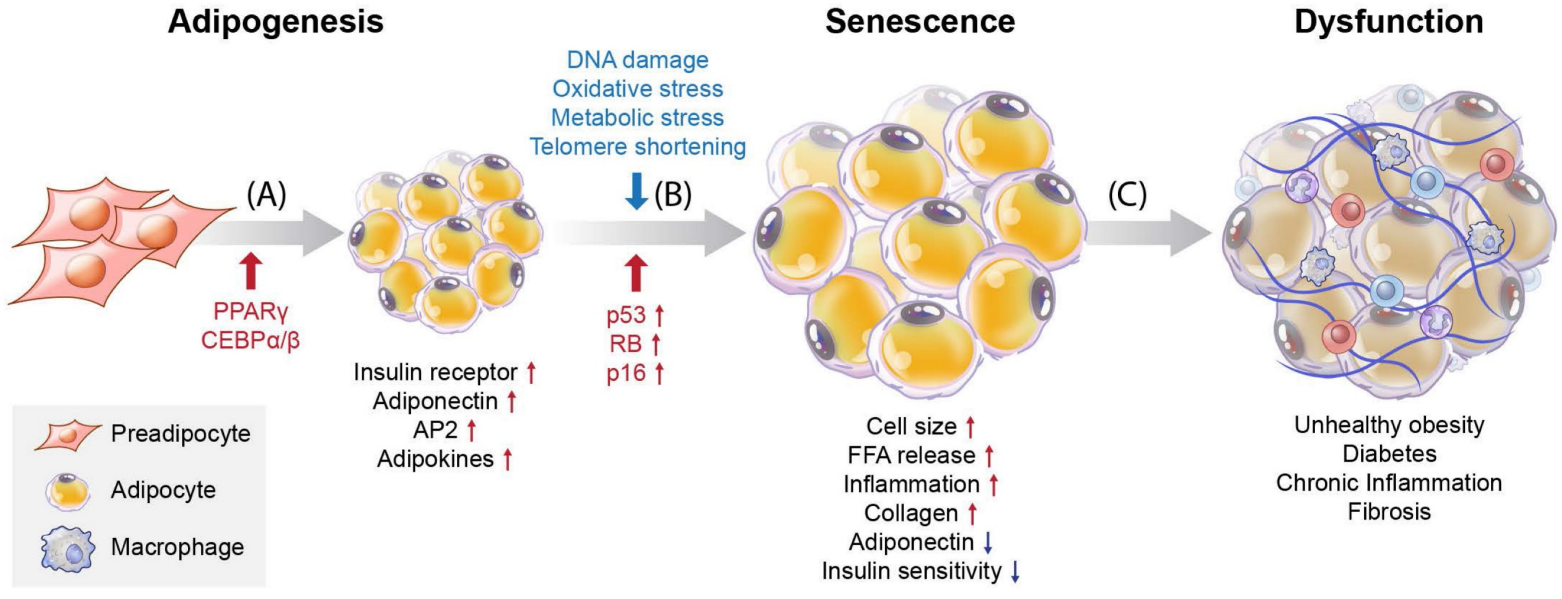
** Overview of differentiation and cellular senescence in adipocytes.** (A) Adipogenesis, (B) senescence, and (C) induction of disease. Pre-adipocytes differentiate into mature adipocytes during adipogenesis. PPARγ and C/EBPα/β are regulators of adipogenesis, and their expression is increased. Also, insulin receptors, adiponectin, aP2, and adipokines are highly expressed in mature adipocytes. Stressors such as hyper-proliferation, DNA damage, oxidative stress, metabolic stress, and telomere shortening activate p53, CDK inhibitors, and RB, which induce senescence. During the accumulation of senescent cells, cell size increases, and free fatty acids (FFAs) are released at high levels with increased inflammation and collagen. Decreased adiponectin and poor insulin sensitivity also occur. These factors are related to cellular dysfunction and cause various diseases such as obesity, diabetes, chronic inflammation, and fibrosis.

Adipogenesis is a key process during cell differentiation from pre-adipocytes to mature adipocytes. Adipocytes proliferate to form adipose tissue, where the energy harvested from food that exceeds the energy expenditure is stored as lipids. Pre-adipocytes develop in four stages: growth arrest, mitotic clonal expansion, early differentiation, and terminal differentiation into mature adipocytes 1. In this process, pre-adipocytes accumulate lipids by increasing the quantities of enzymes needed for triglyceride (TG) synthesis and the accumulation of TG. Increased expression of transcription factors, such as peroxisome proliferator-activated receptor γ (PPARγ), CCAAT/enhancer binding protein α (C/EBPα), and CCAAT/enhancer binding protein β (C/EBPβ), is essential for adipocyte differentiation 2 (Figure 1A). PPARγ is a significant regulator of adipogenesis, and the C/EBP family (α and β) is one of the most critical downstream targets of PPARγ. These proteins are also necessary for the transcription and expression of insulin receptors, adiponectin, adipocyte protein 2 (aP2), and adipokines in mature adipocytes 3, 4.

Understanding the processes involved in adipocyte senescence and their regulation is essential. Adipocyte senescence represents an irreversible cell cycle arrest in response to various stressors, including DNA damage, oxidative stress, metabolic stress, and telomere shortening 5, 6 (Figure 1B). These stressors transmit signals through multiple pathways, most of which activate the cell cycle inhibitor p53. These pathways converge upon the activation of the cyclin-dependent kinase (CDK) inhibitors p16, p21, p27, and p15. Eventually, they activate retinoblastoma protein (RB), causing senescence 7. Unrepaired DNA damage and the loss of repair capacity can induce senescence 8. In adipose tissue, p53 contributes to insulin resistance in age-associated metabolic diseases. Excessive caloric intake exacerbates oxidative stress and increases the production of p53 and pro-inflammatory cytokines in adipose tissue. In contrast, p53 suppression in adipose tissue ameliorates senescence-like changes by reducing pro-inflammatory cytokines and improving insulin sensitivity 9. Interestingly, PPARγ controls adipocyte differentiation, inhibits cellular proliferation, and promotes cellular senescence 10, 11. PPARγ induces the expression of p16^INK4α^ (CDKN2A), a cell cycle inhibitor that promotes senescence, increases senescence-associated-β-galactosidase (SA-β-gal) levels, and triggers G1 arrest 11. Cellular senescence causes functional disorders of adipogenesis and lipid storage in adipose-derived stromal/progenitor cells 12 (Figure 1C).

**Figure 2:**
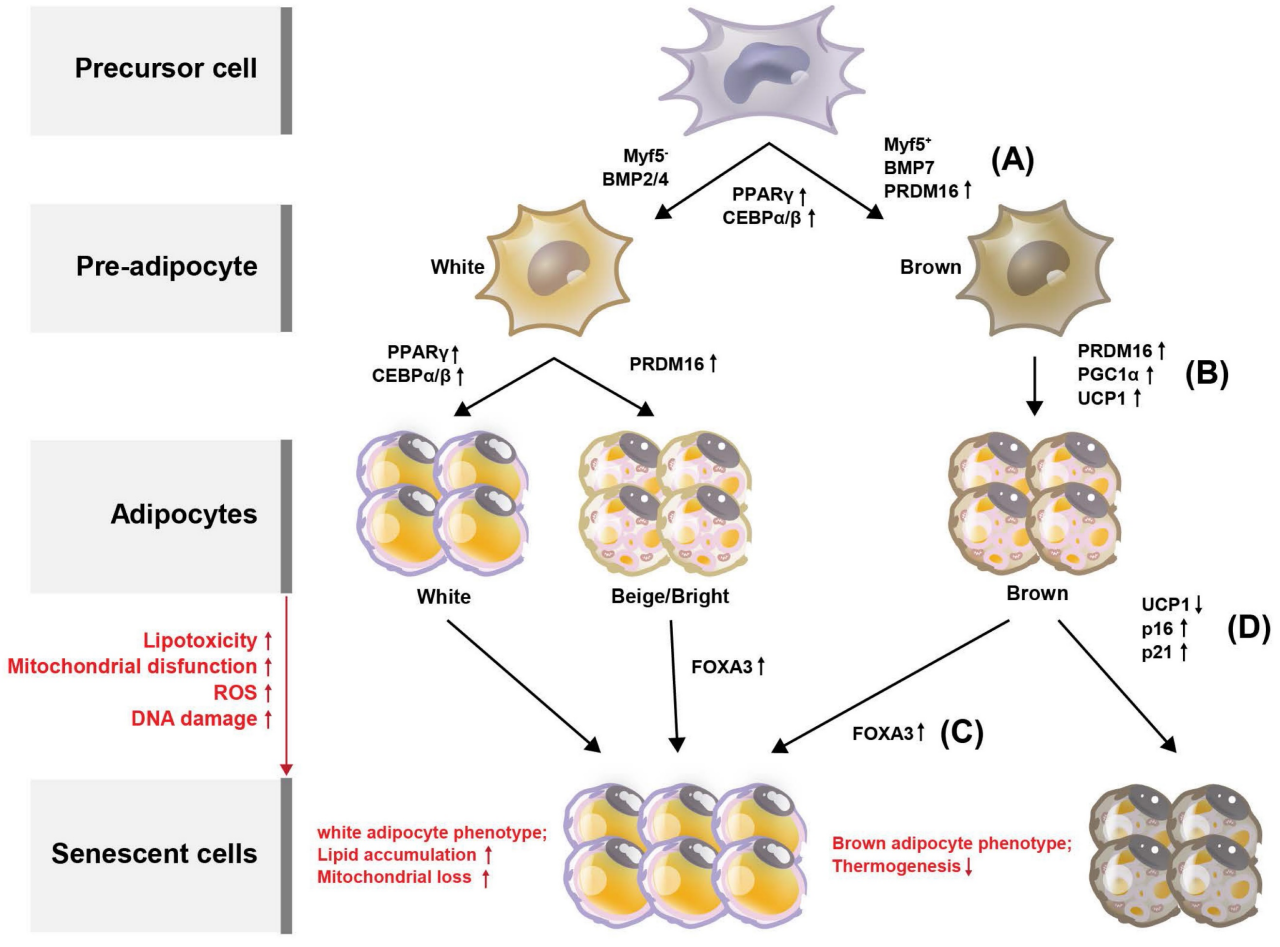
** Processes deciding adipocyte fate during differentiation and senescence, and their regulators in adipose tissues**. Adipose tissue can be divided into white adipose tissue (WAT) and brown adipose tissue (BAT). (A) Through the differentiation of adipocytes, WAT and BAT develop from precursor cells, and Myf5 is expressed from the precursor cells. Pre-adipocytes turn into WAT and BAT during maturation, with transcription factors such as PPARγ, C/EBPs, and PRDM16 regulating this process. Browning of the adipose tissue leads to increased energy expenditure and improved metabolic health. Compared with WAT, BAT contains more mitochondria, which are related to energy production. The increased expression of PPARγ, C/EBPγ, and C/EBPγ is associated with WAT differentiation. (B) BAT is regulated by PGC-1α and PRDM16. Additionally, BAT mitochondria are functionalized by UCP1. With increased lipotoxicity, mitochondrial dysfunction, ROS, and DNA damage, adipocytes become senescent. (C) During senescence, FOXA3 expression is increased concomitant with reduced BAT mass and beiging capacity of WAT. (d) During BAT senescence, mitochondrial enzyme expression is reduced, as is UCP1 expression. In contrast, the expression levels of p16 and p21 are increased.

Browning (or beiging) of adipose tissue refers to the conversion of white adipocytes (WAT) into brown-like adipocytes, such as beige or brite cells (Figure 2). Brown adipocytes (BAT) are thermogenic, meaning they produce heat by burning stored fat. Adipose tissue browning is typically associated with increased energy expenditure and improved metabolic health. BAT, which are responsible for energy production, contain more mitochondria than do WAT which provide energy storage 13. Developmentally, in mice, BAT originates from a myogenic factor 5 (MYF5)-positive mesoderm lineage 14 (Figure 2A). Transcriptional control of the BAT-specific thermogenic program is mediated by PPAR-γ coactivator 1-alpha (PGC-1α) and PR domain-containing 16 (PRDM16) 15 (Figure 2B). It is well established that BAT rely on mitochondrial function for maintaining intracellular metabolism. In addition, BAT mitochondria are functionalized by uncoupling protein-1 (UCP1), which allows the translocation of protons to dissipate energy during non-shivering thermogenesis 16, 17 (Figure 2B). Forkhead box protein A3 (FOXA3) expression is increased in visceral fat during aging and has been reported to reduce BAT mass and the beiging capacity of WAT 18, 19. In the context of aging, it has been reported that mitochondrial enzyme expression is reduced in adipose tissue from old mice, yet little is known regarding the mechanisms that mediate these changes 20, 21. Similarly, it is well-established that human WAT mitochondrial function, as measured by tissue oxygen consumption, is reduced in both obesity and aging 21. Consistent with this, Foxa3-knockout mice are long-lived, have increased BAT activity late in life, and are protected from age-related insulin resistance and high-fat diet-induced increases in visceral adiposity 19 (Figure 2C).

Evidence suggests that replicative capacity and UCP1 expression in BAT are significantly reduced during aging 16, 17. In particular, the proliferative capacity and UCP1 expression in response to cold stimuli appear to be abolished in aged BAT 22. Increasing age of brite adipocytes progressively leads to the development of a WAT phenotype, which prevents adipocyte browning in older mice and humans 23, 24. Conversely, the levels of the senescence markers p16 and p21 are highly increased during the senescence process in BAT 13, 25. BAT thermogenesis declines with aging 26 (Figure 2D), consistent with the reduction in the thermogenic factor UCP1, whereas UCP1 levels in BAT are stabilized by SIRT5 desuccinylation. Sirt5 deficiency in BAT increases the succinylation of UCP1, resulting in decreased UCP1 stability and function, which impairs mitochondrial homeostasis and alters BAT-mediated thermogenesis 27. This may thus be one of the mechanisms regulating BAT senescence.

Some detrimental effects of senescent cell accumulation have been reported, including inflammation, insulin resistance in adipose tissue, underlying obesity, and type 2 diabetes 6, 7, 9, 28. The senescence-associated secretory phenotype (SASP), which includes pro-inflammatory cytokines, is produced via autocrine and paracrine pathways to enhance and diffuse senescent cell influence and induce chronic inflammation in adipose tissues 29. Under stress stimulation conditions, these interactive signaling pathways converge toward the activation of a transcriptional program managed by nuclear factor kappa B (NF-κB) and C/EBPβ, the core effectors that initiate and maintain SASP gene expression 30. Removing senescent adipocytes mitigates inflammation and ameliorates insulin resistance in adipose tissue 29. Therefore, the proper control of adipocyte senescence is critical for the management of various diseases.

The mechanisms controlling cellular senescence can be divided into three categories. These include transcriptional modifications, such as DNA methylation at the genetic and epigenetic levels; mRNA-level regulatory mechanisms, such as mRNA stabilization or degradation; and post-translational modifications (PTMs), such as ubiquitination 31. Here, we focused on PTMs that regulate protein stability and activation. PTMs such as phosphorylation, acetylation, ubiquitination, and glycosylation alter the chemical properties and functions of proteins 32. Abnormal PTMs can cause biological dysfunction and lead to various diseases. For example, PTMs significantly affect the structure and function of proteins that regulate adipocyte senescence. Understanding the role of PTMs in adipocyte senescence may guide the discovery of new therapeutic targets to modulate adipocyte senescence.

**Figure 3:**
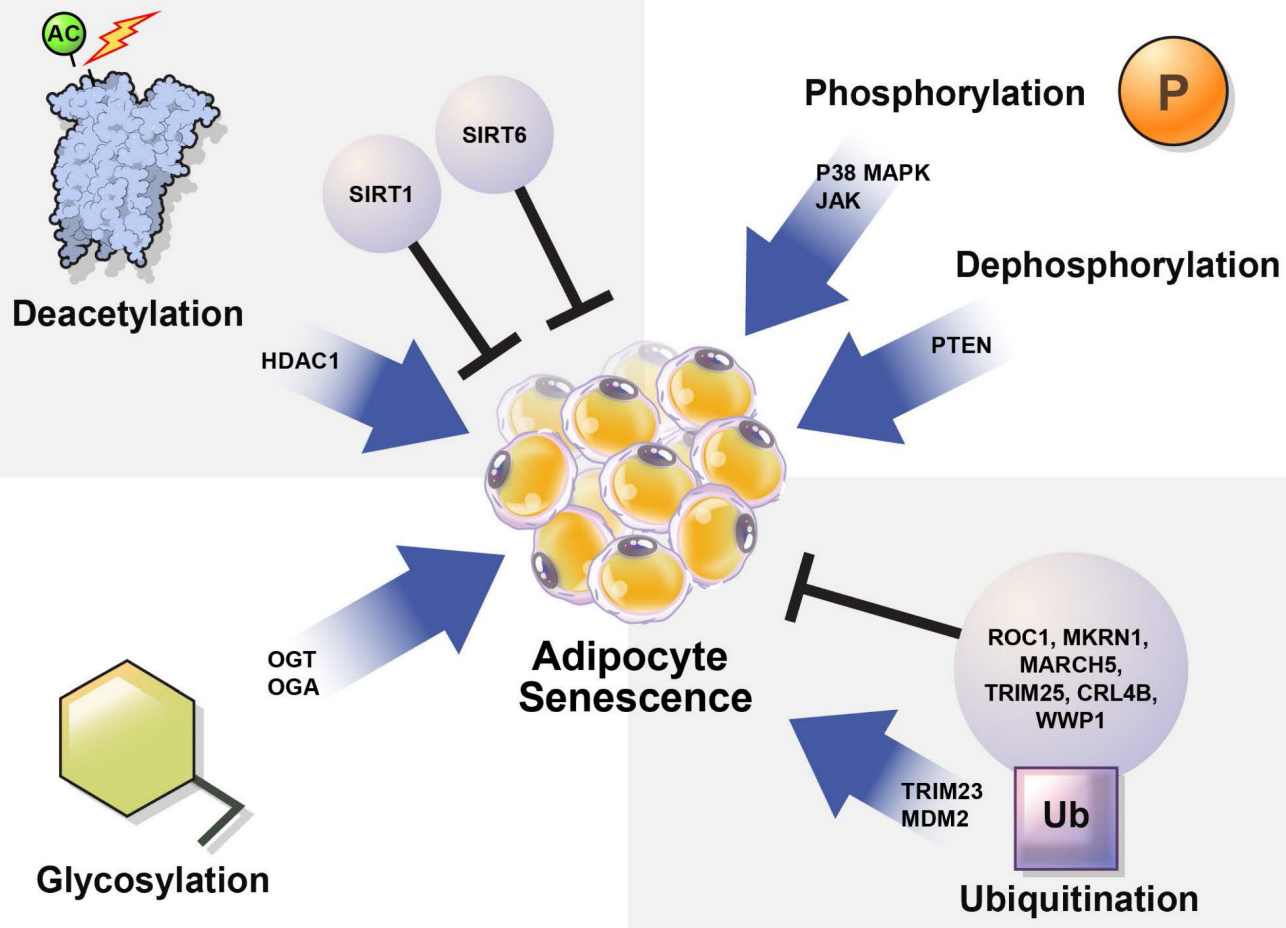
** Regulation of cellular senescence in adipocytes by post-translational modification (PTM).** PTM regulates adipocyte senescence through various physiological pathways, including phosphorylation, deacetylation, ubiquitination, and glycosylation. Inhibition of p38, JAK, and PTEN can alleviate senescence in adipocytes. Deacetylation also regulates adipocyte senescence. SIRT1 and SIRT6 are deacetylases that play protective roles against adipocyte senescence. SIRT1 negatively regulates HDAC1, which is highly expressed in senescent cells. TRIM23 and MDM2 are E3 ubiquitin ligases that induce adipocyte senescence. However, ROC1 suppress cellular senescence. The E3 ubiquitin ligases MKRN1, MARCH 5, TRIM25, CRL4B, and WWP1 are also potential regulators of adipocyte senescence. OGT and OGA regulate *O*-GlcNAcylation. Abnormal protein *O*-GlcNAcylation causes senescence-related diseases.

In this review, we summarize how the stability and activation of proteins involved in the induction of adipocyte senescence are regulated by various PTMs (Table 1) and provide new insights regarding the regulation of adipocyte senescence (Figure 3).

## Regulation of protein activation by phosphorylation

Adipocyte senescence is tightly regulated to maintain energy and metabolic homeostasis 26. Senescent cells accumulate in aging fat in response to replicative, cytokine-induced, and metabolic stresses 33. Janus kinase (JAK), p38, and the phosphatase and tensin homolog on chromosome 10 (PTEN) upregulate adipocyte senescence under various cellular stress conditions. Cellular phosphorylation is known to potentiate or downregulate adipocyte senescence; however, the underlying mechanisms require further investigation.

### JAK

An increase in cellular senescence in response to intrinsic or extrinsic stresses and the broadly related SASP promote organismal aging and adipose tissue dysfunction 26, 34, 35. Inhibition of the JAK/signal transducer and activator of transcription (JAK/STAT) pathway, which plays a significant role in adipose tissue development and function and regulates SASP, can partially inhibit SASP secretion 34, 36. The JAK1/2 inhibitor ruxolitinib decreases the pro-inflammatory SASP *in vitro* and *in vivo* and enhances insulin sensitivity in aging mice 34. Aging cell improvement enhances adipogenesis and metabolism 37, 38. Activin A secreted by senescent cells, blocks adipogenesis. Treatment of aged mice and primary human senescent fat progenitor cells with a JAK inhibitor reduced activin A levels and restored lipid accumulation and expression of critical adipogenic markers. JAK inhibitors also reduce lipotoxicity and increase insulin sensitivity 37. The inhibition of JAK activity is a strategy used to alleviate adipocyte cellular senescence by ameliorating senescent adipose progenitors and stem cells (APSCs).

### p38 MAPK

SASP is potentiated by the activation of p38 mitogen-activated protein kinase (MAPK), which is induced by increased NF-κB transcriptional activity. p38 MAPK inhibition markedly reduced the secretion of most SASP factors 34, 39. SASP contributes to dysfunction in aged organs. Age-related adipose tissue changes increase pro-inflammatory cytokines, such as tumor necrosis factor α (TNF-α), promoting adipocyte senescence 3, 35, 40, which reduces brown adipogenic differentiation and promotes insulin resistance in BATs. During this cellular event, the serine residues of insulin receptor substrate 2 (IRS-2) are phosphorylated by p38 MAPK 40-42. The inhibition of p38 MAPK decreases SASP secretion, which may ameliorate adipocyte senescence.

### PTEN

The insulin signaling response displays heightened sensitivity to cellular aging in adipocytes, thereby decreasing the insulin response 6, 26, 43, 44. PTEN is a phosphatidylinositol phosphate phosphatase that plays an essential role in various cellular processes, including genome maintenance, DNA repair, cell cycle control, proliferation, metabolism, migration, tumorigenesis, and senescence. One of the critical roles of PTEN is to inhibit insulin signaling 45-48. PTEN negatively regulates insulin signaling by dephosphorylating phosphatidylinositol-3,4,5-triphosphate (PIP3), resulting in decreased phosphatidylinositol 3-kinase/protein kinase B (PI3K/AKT) signaling 49. Pten deficiency enhances energy expenditure in brown adipose tissue. PTEN loss and activation of PI3K/AKT signaling lead to an improved ability to handle metabolic stress in mice 50. PTEN downregulation increased the proliferation of stromal vascular fraction (SVF) cells, including adipocyte progenitor cells, in adipose tissues. Pten deficiency restores the differentiation capacity of high-passage SVF cells and increases adipogenesis. In contrast, PTEN expression is upregulated during cellular senescence 51. The regulation of PTEN activity and expression levels is a strategy for controlling adipocyte cellular senescence.

## Regulation of protein activation by deacetylation

Senescent adipocytes accumulate in aging fat in response to cytokines and metabolic stress 26, 33. Adipocyte senescence is regulated in response to cold exposure-stimulated thermogenesis. The mitochondrial function and activity of UCP-1, a thermogenesis-related mitochondrial protein in adipocytes, decrease with cellular aging. Additionally, the pro-inflammatory capacity of BAT increases with age 26, 35, 52. Histone deacetylases (HDACs) such as sirtuin 1 (SIRT1), SIRT 6, and HDAC1 are essential regulators of adipocyte senescence under conditions of cellular stress 26, 53, 54. Although SIRTs and HDAC1 are deacetylases, their roles in adipocyte senescence differ. SIRT1 and SIRT6 prevent adipocyte senescence, whereas HDAC1 potentiates adipocyte senescence. The number of brown and beige adipocytes decreases with age. Cellular deacetylation events regulate adipocyte senescence. However, the underlying mechanisms remain unclear.

### SIRT1

A reduction in beige adipocyte formation has been detected in aging adipose tissues 26. SIRT1, a nicotinamide adenine dinucleotide (NAD^+^)-dependent deacetylase, drives beige adipocyte generation in WAT 26. In addition, NAD^+^ levels, SIRT1 activity, and SIRT1 expression decrease with age and cellular senescence progression 10, 55, 56. PPARγ directly interacts with and negatively regulates SIRT1 expression 10. PPARγ acetylation, which is correlated with Sirt1 deficiency, increases during cellular senescence 10. SIRT1 limits pre-adipocyte hyperplasia through c-Myc deacetylation, improves insulin sensitivity, reduces inflammation, and suppresses lipid accumulation by inhibiting PPARγ 57. The proto-oncoprotein zinc finger and BTB domain-containing 7C (ZBTB7C) negatively regulate Sirt1 transcript levels. Its expression level is increased in the WAT of aging mice. ZBTB7C, a potent SIRT1 repressor, increases PPARγ acetylation 58. Expression of nicotinamide phosphoribosyltransferase (NAMPT), which recycles NAD^+^, increases during cellular senescence. NAMPT activity promotes pro-inflammatory SASP 34, 59-61. Age-related reduction in SIRT1 activity may be a critical mechanism in the loss of beige adipose tissue as well as in age-associated thermogenic impairment 26. Upregulation of SIRT1 potentiates brown remodeling of subcutaneous WAT by deacetylation of PPARγ at Lys268 and Lys293 62. SIRT1 regulation is likely a key mechanism controlling adipocyte senescence.

### SIRT6

The essential roles of SIRT6 in adipocytes are the regulation of lipid metabolism and the prevention of inflammation. SIRT6 stimulates lipolysis, enhances adipose tissue browning, and ameliorates adipose tissue inflammation, thereby improving insulin action in the peripheral tissues 63. SIRT6 promotes cell proliferation and antagonizes cellular senescence; however, SIRT6 expression decreases during cellular senescence 64. Moreover, SIRT6 suppresses p27^Kip1^ (p27) expression during cellular senescence 64. SIRT6 mediates the polyubiquitination of p27, directing its degradation by the proteasome and thereby regulating the acetylation status of p27. Thus, SIRT6 delays cellular senescence 64. Aging and excessive caloric intake, which are two major risk factors for obesity and diabetes, lead to decreased SIRT6 levels 65. Sirt6 deficiency in pre-adipocytes blocks adipogenesis and regulates mitotic clonal expansion 66. Sirt6 deletion in adipose tissue impairs the thermogenic function of BAT, causing morphological ''whitening'' of brown fat, reduced oxygen consumption, obesity, decreased core body temperature, and cold sensitivity. Fat Sirt6-deleted mice exhibit increased blood glucose levels, severe insulin resistance, and hepatic steatosis. Moreover, Sirt6 deficiency inhibits WAT browning following cold exposure or β3-agonist treatment 67. Taken together, these results indicate that SIRT6 plays a protective role against adipocyte senescence.

### HDAC1

Inhibition of signaling pathways that induce SASP using HDAC inhibitors, including trichostatin A (TSA), suppresses senescence. At low concentrations, TSA acts as a pan-SASP blocker 34, 68. TSA may decrease PPARγ expression 53, 69, potentiating cellular senescence. HDAC inhibitors are also involved in regulating thermogenic adipocyte differentiation, adaptive thermogenesis, and metabolic disorder pathogenesis 70. HDAC1 is highly expressed in senescent cells 71. HDAC1 negatively regulates the thermogenic program in BAT. Repression of HDAC1 promotes acetylation and prevents methylation of histone H3K27, which increases the expression of BAT-specific genes such as UCP1, PGC-1α, and PRDM16 70, 72. SIRT1 negatively regulates HDAC1 function 54, 73. SIRT1 is degraded and downregulated during cellular senescence 56, 74, indicating that Sirt1 deficiency may increase HDAC1 activity in senescent cells 54. Thus, regulation of HDAC1 function may improve adipocyte senescence.

## Regulation of protein stability by ubiquitination: E3 ubiquitin ligases

Another regulatory mechanism that influences physiological cellular senescence is the post-translational modification of cellular proteins through ubiquitination. The ubiquitin-proteasome pathway (UPP) regulates the differentiation of various cell types. Alterations in the UPP in mature adipocytes can potentially modulate adipose function during adipocyte aging 75. Ubiquitin is activated by E1 and transferred to E2 or Ub conjugase. In turn, the E2 enzyme transfers ubiquitin to E3 or directly ubiquitinates the target protein in conjunction with E3 72, 73. Proteasome activity decreases during senescence, which may be associated with aging and age-associated diseases 75. E3 ubiquitin-protein ligases are crucial factors in the regulation of senescence by ubiquitination. Knockdown of regulator of cullins-1 (ROC1), a component of the SKP, Cullin, F-box (SCF) E3 ubiquitin ligases, suppresses the growth of several human cell lines by inducing senescence 76. In addition, the inactivation of the von Hippel-Lindau (VHL) tumor suppressor gene, which encodes a subunit of an E3 ubiquitin ligase, causes a senescence-like phenotype in human cancer cell lines 77. Therefore, E3 ubiquitin ligase activity appears to be essential for regulating adipocyte senescence. Several E3 ligases have recently been found to be present in adipocytes, including seven in absentia homolog 2 (SIAH2), makorin ring finger protein 1 (MKRN1), tripartite motif protein 23 (TRIM23), and neural precursor cell-expressed developmentally downregulated protein 4 (NEDD4) 78. The ubiquitination pathway regulates p53 tumor suppressor stability, localization, and functions in normal cells 79. E3 ubiquitin ligases, including murine double minute 2 (MDM2) and MKRN1, predominantly regulate p53 expression levels and activities under various physiological conditions 79, 80. Overexpression of MDM2 and CDK4 can induce human telomere reverse transcriptase (hTERT) overexpression and p53 degradation in human 2H transgenic bone marrow-mesenchymal stem cells (BM-MSCs), increase cell proliferation and migration, and suppress the adipogenic differentiation potential *in vitro* 81.

### MKRN1

In telomerase-positive cells, overexpression of MKRN1, an E3 ligase, promotes hTERT degradation, decreases telomerase activity, and subsequently decreases telomere length 82. MKRN1 knockdown induces senescence by stabilizing p14^ARF^. MKRN1 also regulates SASP 83. MKRN1 negatively regulates PPARγ via ubiquitin-mediated proteasomal degradation, with PPARγ2 and MKRN1 interacting directly 84. MKRN1 also ubiquitinates and degrades AMP-activated protein kinase alpha (AMPKα) subunits, whereas MKRN1 depletion promotes glucose consumption and suppresses lipid accumulation via AMPK stabilization and activation 85. These results suggest that the E3 ligase MKRN1 potentially regulates adipocyte senescence, warranting further studies.

### TRIM23 and TRIM25

TRIM23 is an E3 ligase that can regulate PPARγ protein stability and mediate abnormal polyubiquitin conjugation 86. TRIM23 is required to form late enhanceosomes and recruit Pol II during late adipogenic differentiation, whereas treatment with the proteasome inhibitor MG132 inhibits the reduction of PPARγ in TRIM23-knockdown cells 86. The 26S proteasome does not readily recognize PPARγ aberrantly ubiquitinated by TRIM23, resulting in its protection from proteasomal degradation 86. However, TRIM25 directly induces PPARγ ubiquitination and its proteasome-dependent degradation 87. TRIM25 decreases PPARγ expression and inhibits 3T3-L1 adipocyte differentiation 87. Therefore, TRIM25 expression is negatively correlated with PPARγ expression.

### MARCH 5

The E3 ubiquitin ligase, membrane-associated RING-CH-type finger 5 (MARCH 5) regulates mitochondrial dynamics and is in turn regulated by PPARγ in adipocytes undergoing adipogenesis 88. MARCH 5 depletion increases glycolysis and basal mitochondrial respiration 88. MARCH5-deficient cells display mitochondrial elongation and phenotypic changes owing to increased SA-β-Gal expression caused by cellular senescence 89.

### CRL4B

The aryl hydrocarbon receptor (AhR) reduces PPARγ protein stability through a proteasome-dependent mechanism 90. Overexpression of AhR in 3T3-L1 cells induced a decrease in endogenous PPARγ, which was reversed by treatment with MG132 90. AhR serves as a substrate receptor in the Cullin 4B-RING E3 ubiquitin ligase (CRL4B) AhR complex to induce polyubiquitination of PPARγ 90.

### MDM2

MDM2, an E3 ubiquitin ligase, regulates adipogenesis by initiating adipocyte differentiation through the promotion of cAMP-mediated transcriptional activation of cAMP response element-binding proteins (CREB) and the induction of C/EBPδ expression 91. High-fat diet (HFD)-fed Mdm2-adipocyte-specific knock-in (Mdm2-AKI) mice display epididymal white adipose tissue (eWAT) dysfunction, including senescence 92. MDM2 suppresses six-transmembrane epithelial antigen of prostate 4 (STEAP4) expression via ubiquitin modification 92. Revival of STEAP4 rescued MDM2-induced adipose dysfunction in eWAT of HFD-fed Mdm2-AKI mice 92.

### WWP1

Obesity upregulates WW domain-containing E3 ubiquitin protein ligase 1 (WWP1) in WAT. WWP1, which belongs to the NEDD4-like family of E3 ubiquitin ligases, is upregulated in obese WAT 93. WWP1 induces p27^Kip1^ degradation via ubiquitination and inhibits p27^Kip1^-mediated replicative senescence 94. WWP1 overexpression decreases reactive oxygen species (ROS) levels in 3T3-L1 cells, and WWP1 protects against obesity-associated oxidative stress in adipocytes and WAT 95.

## Regulation of protein activation by O-GlcNAcylation

*O*-linked-N-acetylglucosamination (*O*-GlcNAcylation), a post-translational glycosylation event, occurs on proteins in the nucleus, cytoplasm, and mitochondria, regulates cell signaling, and is associated with several pathological conditions 96. *O*-GlcNAcylation is the single-sugar addition of *O*-linked-β-N-acetylglucosamine (*O*-GlcNAc) to the hydroxyl groups of the serine or threonine residues of target proteins 97. The attachment and removal of *O*-GlcNAc from proteins are processed by *O*-GlcNAc transferase (OGT) and *O*-GlcNAcase (OGA) 98. *O*-GlcNAcylation is highly responsive to glucose, and insulin resistance enhances *O*-GlcNAcylation 99. Increased levels of *O*-GlcNAcylation have been observed in response to the induction of adipocyte differentiation 100. Post-translational modifications regulate OGT activity, and OGT activation occurs when tyrosine phosphorylation of OGT increases following insulin stimulation in 3T3-L1 cells 101. Abnormal protein *O*-GlcNAcylation is essential for the development and progression of senescence-related diseases 102.

*O*-GlcNAcylation levels increased significantly on and after day 5 of 3T3-L1 differentiation induction 100. Simultaneously, C/EBPα and adiponectin expression, and lipid droplet size increased 100. *O*-GlcNAcylation of vimentin, long-chain fatty acid-CoA, and pyruvate carboxylase increases with adipocyte differentiation 100. Treatment with 6-diazo-5-oxo-norleucine (DON), a glutamine:fructose-6-phosphate amidotransferase (GFAT) inhibitor, blocked adipocyte differentiation at the stage of C/EBPα expression, which was associated with an overall increase in *O*-GlcNAcylation 100. Therefore, it can be assumed that *O*-GlcNAcylation partially participates in adipogenesis.

In 3T3-L1 adipocytes, *O*-GlcNAc causes post-transcriptional modification of PPARγ. The primary *O*-GlcNAc site of PPARγ is threonine 54 of the N-terminal activation function-1 domain 97. In 3T3-L1 cells, an increase in *O*-GlcNAc modification mediated by the OGA inhibitor NButGT decreases PPARγ transcriptional activity and terminal adipocyte differentiation 97.

C/EBPβ is also modified by *O*-GlcNAc, which is present in nucleocytoplasmic proteins. GlcNAcylation sites (Ser^180^ and Ser^181^) are located in the regulatory domain and are extremely close to the phosphorylation sites (Thr^188^, Ser^184^, and Thr^179^) that regulate DNA-binding activity 102. GlcNAcylation of Ser^180^ and Ser^181^ blocks the phosphorylation of Thr^188^, Ser^184^, and Thr^179^, thereby delaying adipocyte differentiation 102. In contrast, the mutation of Ser^180^ and Ser^181^ to Ala increased C/EBPβ transcriptional activity 102. Therefore, GlcNAcylation and phosphorylation appear to modulate the function of C/EBPβ by alternately occupying adjacent sites.

The hexosamine biosynthesis pathway (HBP) flux functions as a nutrient sensor and induces *O*-GlcNAc modification of the AMPK α subunit in both immortal and primary murine adipocytes 103. *O*-linked glycosylation via HBP flux regulates AMPK activation and induces fatty acid oxidation in 3T3-L1 adipocytes 104 whereas removal of *O*-GlcNAc by hexosaminidase reduces AMPK activity 104. Thus, HBP correlates with *O*-GlcNAc and is likely to affect adipocyte senescence.

## Conclusions and perspectives

Adipogenesis is the process of differentiation of pre-adipocytes into mature adipocytes. There are two main types of fat cells that contain lipids: WAT, which store energy, and BAT, which produces heat. Pre-adipocytes develop in four stages: growth arrest, mitotic clone expansion, early differentiation, and terminal differentiation into mature adipocytes 1. In this process, increased expression of transcription factors is essential for adipocyte differentiation 2 (Figure 1A).

Upon DNA damage, oxidative stress, metabolic stress, or telomere shortening, adipocytes undergo senescence and irreversible cell cycle arrest, thereby inhibiting adipogenesis 5, 6. These cellular stressors activate p53 and induce CDK inhibitors and RB, which cause senescence 7. In addition, the modification of adipogenesis regulators such as PPAR and C/EBPα/β results in adipocyte senescence. Moreover, mitochondrial UCP1 expression has been reported to decrease in old mouse adipocytes; however, little is known about this mechanism 16, 17, 27. As UCP1 levels decrease during the aging of BAT, the levels of aging markers p16 and p21 increase significantly 13, 25. In addition, the expression of UCP1, a thermogenesis factor, in BAT is stabilized by SIRT5 desuccinylation. This may thus be a mechanism by which BAT senescence is regulated; however, this possibility requires further investigation.

Once lipids continuously accumulate in the adipose tissue, the adipocyte senescence rate increases and insulin sensitivity decreases, resulting in adipose tissue dysfunction. SASP induces chronic inflammation in adipose tissue. Cellular senescence causes lipid storage dysfunction. Thus, the appropriate control of fat cell aging is a viable strategy for preventing aging-related diseases. Although the underlying mechanisms remain poorly understood, adipocyte senescence is essential for diverse physiological processes, including metabolism and various age-related diseases. To better understand the processes of adipocyte senescence, it is important to identify the modulators of adipogenic factors, including PPARγ, and their regulatory molecular mechanisms, such as PTMs. PTMs are associated with oxidative stress, inflammation, and aging, thereby influencing aging characteristics 105. Some PTMs participate in healthy aging, suggesting that they are essential regulators and predictive markers of the senescence process 106. PTMs significantly affect aging by targeting epigenetic and non-epigenetic pathways. Therefore, understanding the role of PTMs in cellular senescence may advance the development of targeted therapies for age-related diseases.

p38, JAK, and PTEN influenced fat cell senescence through phosphorylation under stress (Figure 3). Inhibition of the JAK/STAT pathway inhibits SASP secretion and Pten deficiency increases adipogenesis 34, 36-39.

SIRT1 induces beige adipocyte production in WAT 107. PPARγ interacts directly with SIRT1 to negatively regulate SIRT1 expression 10. NAMPT activity that rescues NAD^+^ promotes SASP. SIRT6 also inhibits p27 expression during cell aging, thereby slowing this process 34, 59-61. SIRT1 and SIRT6 regulation is expected to be a key mechanism in controlling fat cell aging 63-65, 67. Suppression of the SASP pathway inhibits aging. HDAC1 is highly expressed in older cells. Regulation of HDAC1 function may retard adipocyte aging (Figure 3).

The inactivation of tumor suppressor genes can result in phenotypes similar to those observed during aging 77. Therefore, E3 ubiquitin ligase activity appears to be essential for the regulation of adipocyte aging. MKRN1 may negatively regulate PPARγ through ubiquitination 84. Because TRIM25 reduces PPARγ expression and suppresses the differentiation of 3T3-L1 adipocytes, TRIM25 correlates negatively with PPARγ expression 87. MARCH 5 depletion increases glycolysis and basal mitochondrial respiration 88. In addition, components of E3 ubiquitin ligases such as ROC1, TRIM23, CRL4B, WWP1, and MDM2 inhibit adipocyte senescence 76, 86, 90, 92, 95.

O-GlcNacylation regulates cell signaling and plays an essential role in the development and progression of age-related diseases. OGT and OGA regulate the attachment and removal of O-GlcNAc. In 3T3-L1 cells, these enzymes induce adipocyte senescence. We predict that new treatments for adipocyte senescence can be developed by understanding the involvement of PTMs in fat-cell aging (Figure 3).

In particular, a clearer understanding is needed regarding how PTMs, including acetylation/deacetylation, phosphorylation, ubiquitination, and glycosylation, regulate the function of necessary adipogenic factors, such as PPAR (Figure 3). Finally, it is important to establish the degree to which prevention of adipocyte senescence mediates beneficial effects on adipose tissue in various disease conditions. Future research on the regulatory mechanisms underlying adipocyte senescence will likely provide critical insights regarding the new and complex networks involved in human biological processes, including aging and metabolism.

## Figures and Tables

**Table 1:** The summary of Post-translational Modification (PTM) in adipocyte senescence

PTM		Substrate	Pathway	Action site
Phosphorylation	JAK^34^, ^36^, ^37^	SASP	JAK/STAT pathway	
	P38 MAPK^40^-^42^	IRS-2	p38 MAPK signaling pathway	
Dephosphorylation	PTEN^49^-^51^	PIP3	PI3K/AKT signaling pathway	D3
Deacetylation	SIRT1^57^,^58^,^62^	PPARγ	Lipogenesis	Lys^268^ and ^Lys293^
	SIRT6^64^	p27^Kip1^	Adipocyte differentiation	Lys^100^
	HDAC1^70^, ^72^	H3K27	Thermogenesis	Lys^27^
Ubiquitination	MKRN1^83^-^85^	p14^ARF^, AMPKα	Fatty acid oxidation	
	TRIM 23^86^	PPARγ	Adipocyte differentiation	
	TRIM 25^87^			
	MARCH 5^88^		Glycolysis and basal mitochondrial respiration	
	CRL4B^90^	PPARγ	Adipocyte differentiation	
	MDM2^92^	STEAP4	HIF1-α/PKM2 signaling pathway	Lys^18^ and Lys^161^
	WWP1^94^	p27^Kip1^	Adipocyte differentiation	
O-GlcNAcylation	OGT, OGA^97^, ^100^,^102^	PPARγ,	Adipocyte differentiation	Thr^54^ of the N-terminal activation function-1 domain
		C/EBPβ		Ser^180^ and Ser^181^
	HBP flux^103^,^104^	AMPKα	Fatty acid oxidation	-

## Abbreviations

AhR: aryl hydrocarbon receptor
AMPK: AMP-activated protein kinase
aP2: adipocyte protein 2
BAT: brown adipocyte
CDK: cyclin-dependent kinase
C/EBPα/b: CCAAT/enhancer binding protein α/b
CREB: cAMP-mediated transcriptional activation of cAMP response element-binding proteins
CRL4B: Cullin 4B-RING E3 ubiquitin ligase
eWAT: epididymal white adipose tissue
FOXA3: forkhead box protein A3
HBP: hexosamine biosynthesis pathway
HDAC: histone deacetylase
HFD: high-fat diet
hTERT: human telomere reverse transcriptase
JAK/STAT: Janus kinase/signal transducer and activator of transcription
MAPK: mitogen-activated protein kinase
MARCH5: membrane-associated RING-CH-type finger 5
MDM2: murine double minute 2
MKRN1: makorin ring finger protein 1
MYF5: myogenic factor 5
NAD: nicotinamide adenine dinucleotide
NAMPT: nicotinamide phosphoribosyltransferase
NEDD4: neural precursor cell-expressed developmentally downregulated protein 4
NF-κB: nuclear factor kappa B
OGA: *O*-GlcNAcase
*O*-GlcNAc: *O*-linked-β-N-acetylglucosamine
*O*-GlcNAcylation: *O*-linked-N-acetylglucosamination
OGT: *O*-GlcNAc transferase
p27: p27^Kip1^
PGC-1α: PPAR-g coactivator 1-alpha
PI3K/AKT: phosphatidylinositol 3-kinase/protein kinase B
PPARγ: peroxisome proliferator-activated receptor γ
PRDM16: PR domain-containing 16
PTEN: phosphatase and tensin homolog on chromosome 10
PTM: post-translational modifications
RB: retinoblastoma protein
ROC1: regulator of cullins-1
SA-β-gal: senescence-associated-β-galactosidase
SASP: senescence-associated secretory phenotype
SIRT: sirtuin
STEAP4: six-transmembrane epithelial antigen of prostate 4
SVF: stromal vascular fraction
TG: triglyceride
TRIM23: tripartite motif protein 23
TSA: trichostatin A
UCP1: uncoupling protein-1
UPP: ubiquitin-proteasome pathway
WAT: white adipocyte
WWP1: WW domain-containing E3 ubiquitin protein ligase 1
ZBTB7C: zinc finger and BTB domain-containing 7C
